# Zoledronate Extends Health Span and Survival via the Mevalonate Pathway in a FOXO-dependent Manner

**DOI:** 10.1093/gerona/glab172

**Published:** 2021-06-17

**Authors:** Zhengqi Chen, Julia Cordero, Adel M Alqarni, Cathy Slack, Martin P Zeidler, Ilaria Bellantuono

**Affiliations:** Healthy Lifespan Institute, Department of Oncology and Metabolism, The Medical School, University of Sheffield, UK; Institute of Cancer Sciences, University of Glasgow, Beatson Institute for Cancer, UK; Department of Biomedical Science, University of Sheffield, UK; School of Life & Health Sciences, Aston University, Birmingham, UK; Department of Biomedical Science, University of Sheffield, UK; Healthy Lifespan Institute, Department of Oncology and Metabolism, The Medical School, University of Sheffield, UK

**Keywords:** Aging, Bisphosphonates, DNA damage, *Drosophila*, Life span

## Abstract

Over recent decades, increased longevity has not been paralleled by extended health span, resulting in more years spent with multiple diseases in older age. As such, interventions to improve health span are urgently required. Zoledronate (Zol) is a nitrogen-containing bisphosphonate, which inhibits the farnesyl pyrophosphate synthase enzyme, central to the mevalonate pathway. It is already used clinically to prevent fractures in osteoporotic patients, who have been reported to derive unexpected and unexplained survival benefits. Using *Drosophila* as a model we determined the effects of Zol on life span, parameters of health span (climbing ability and intestinal dysplasia), and the ability to confer resistance to oxidative stress using a combination of genetically manipulated *Drosophila* strains and Western blotting. Our study shows that Zol extended life span, improved climbing activity, and reduced intestinal epithelial dysplasia and permeability with age. Mechanistic studies showed that Zol conferred resistance to oxidative stress and reduced accumulation of X-ray-induced DNA damage via inhibition of farnesyl pyrophosphate synthase. Moreover, Zol was associated with inhibition of phosphorylated AKT in the mammalian traget of rapamycin pathway downstream of the mevalonate pathway and required dFOXO for its action, both molecules associated with increased longevity. Taken together, our work indicates that Zol, a drug already widely used to prevent osteoporosis and dosed only once a year, modulates important mechanisms of aging. Its repurposing holds great promise as a treatment to improve health span.

It has been estimated that by 2050, Europe, North America, and 8 countries across the other continents will have more than 30% of their population over the age of 60 (1). However, this increase in life expectancy has not translated into extended health span (2). More than 60% of those over the age of 65 suffer from multimorbidities—defined as the coexistence of multiple chronic health conditions (3). These multimorbidities impose quality-of-life and care management challenges associated with treating multiple conditions individually and are frequently associated with increased costs, reduced efficacy, and increased likelihood of adverse events due to polypharmacy (4). Targeting pathways underpinning central mechanisms of aging, which are common to multiple disorders, offers new opportunities to treat multimorbidity and overcome these problems.

Zoledronate (Zol) is a nitrogen-containing bisphosphonate used for the treatment of skeletal disorders, including osteoporosis. It inhibits the mevalonate pathway through inhibition of the enzyme farnesyl pyrophosphate synthase (FPPS) (5) and thereby inhibits bone resorption by inhibition of osteoclast activity. Due to its high-affinity binding to hydroxyapatite crystal mineralization, Zol is strongly enriched in bone and released following bone breakdown conferring long-lasting activity (6). In clinical practice, Zol is administered by intravenous infusion once a year in postmenopausal women to prevent fractures (7,8). Recently, retrospective analysis of several clinical trials showed that patients treated with Zol had a significant decrease in mortality rates (9). In addition, among patients admitted to intensive care units, those previously treated with Zol had increased survival despite overall higher level of multimorbidities and older age (10). It is unknown whether other mechanisms independent of bone protection may be involved in the Zol-dependent extension of survival.

The mevalonate pathway is an important metabolic pathway responsible for the production of cholesterol and protein prenylation. One key group of isoprenylated proteins are small GTPases (11), important signaling molecules which play central roles in multiple cellular processes, including cellular morphology, integrin function, and longevity-associated pathways such as mammalian traget of rapamycin (mTOR) (12). In this study we examined whether Zol is able to extend life span and health span independent of its effect on bone using *Drosophila*, a model widely used for aging studies. While retaining evolutionarily conserved components of both the mevalonate and mTOR pathways, *Drosophila* does not feature the bone-like mineralization present in mammalian models (13). This approach allows us to exclude mechanisms, which have been hypothesized to explain Zol-mediated increases in human survival. These include the bone acting as a regulator of other tissues’ homeostasis (10) through the release of hormones such as osteocalcin, a regulator of glucose homeostasis (14). In doing so, our approach allows osteoprotective and geroprotective activities to be dissected in vivo. Our results uncovered a previously unrecognized role of Zol extending both life span and health span and improving survival in the presence of oxidative stress. The extension in survival by Zol is associated with a reduction in phosphorylated AKT (pAKT) expression and requires dFOXO signaling, a conserved pathway well known for its positive effects on longevity.

## Experimental Methods

### Fly Stocks and Husbandry

The *white*^*Dahomey*^ (*w*^*Dah*^) stock has been maintained in large population cages with overlapping generations since 1970 (15) and was a kind gift of the Partridge lab. *y*^*1*^*,w*^*67c23*^*; P(lacW)Fpps*^*k06103*^*/CyO* and *y*^*1*^*,w*^*67c23*^*; raw*^*k03514*^*, P(lacW)Fpps*^*k03514*^*/CyO* (16) and *w*^*1118*^*; foxo*^*Δ94*^*/TM6B, Tb*^*1*^ (17) were obtained from the Bloomington *Drosophila* Stock Centre. *w; Su(H)-lacZ, esg-Gal4,UAS-GFP/CyO* expresses green fluorescent protein (GFP) in intestinal stem cells and progenitor cells and was a kind gift of Leanne Jones (18). *w*^*+*^*; GMR-Gal4,UAS-white*^*RNAi*^ is a recombinant between *GMR-Gal4* (19) and GD30033, an in vivo hairpin-loop RNAi construct targeting *white* mRNA from the Vienna *Drosophila* Resource Center (20).

All flies were maintained in a 12:12-h light-dark cycle on standard yeast, molasses, cornmeal, agar food at 18 or 25°C. Drug-containing food was prepared using freshly cooked molten fly food cooled to 60°C. Zol (kindly provided by Hal Hebetino, University of Rochester), geranyl geraniol (GGOH; Sigma Aldrich, Dorset, United Kingdom), and farnesyl farnesol (FOH; Sigma Aldrich) or carrier(s) were added, mixed thoroughly, and immediately poured into standard vials and bottles so as to minimize exposure of drug to high temperatures. The continued activity of Zol following brief exposure to this temperature was confirmed molecularly (data not shown). Drug food was stored at 4°C until use for a maximum of 3 weeks.

For life-span experiments overnight embryo collections from approximately 200 adult flies were collected on apple juice agar plates, washed with water, and 32 µL of embryos transferred into food bottles. Flies that subsequently ecclosed within the first 24 hours of the first to emerge were discarded with those subsequently ecclosing over a 16- to 20-hour overnight collection window being used for experiments. These later flies were transferred into new food bottles without the use of CO_2_ and incubated for 3–4 days. Flies were then sorted by gender and counted while minimizing CO_2_ exposure.

### Longevity Assay and H_2_O_2_ Survival Assays

For longevity assays, 20 gender-matched flies were maintained in vials of food and transferred into new vials every 2–3 days. At every transfer numbers of dead or censored flies were recorded. Unless specifically specified 100 flies were analyzed for each experimental with 3 independent experimental replicates. To test survival in presence of hydrogen peroxide, adult flies of the appropriate genotype and pretreatment were first starved in vials containing 1% agar for 3 hours and then transferred to standard food containing 5% H_2_O_2_ and the appropriate drugs/vehicle. Death was then scored at 12, 24, and 36 hours and then every 2 hours for the next 48 hours. If 100% mortality was not reached by that point, scoring was continued every 12 hours.

### Rapid Iterative Negative Geotaxis Assays

For rapid iterative negative geotaxis assay flies were kept in groups of ~150. The day before an experiment, flies were sorted into groups of 20 and transferred into separate vials following brief CO_2_ anesthesia. Flies were then transferred into 25-mL strippette (Fisher Scientific) and after 1 minute, the strippette was tapped to knock flies to the bottom and initiate the negative geotaxic response. After 15 seconds a photograph was taken and the position of each fly, and hence the distance climbed, was recorded. Twenty flies were assessed from each condition with each experiment repeated 3 times. Flies that climbed above 10 cm were classified as “high climbers.”

### Gut Barrier Integrity and Food Uptake Assays

For gut barrier integrity assays *Drosophila* food containing 2.5% (w/v) erioglaucine disodium salt (Sigma Aldrich) and drugs (as appropriate) was fed to flies for 9 hours. For gut integrity assays flies were scored for uniform blue coloration beyond the gastrointestinal tract (“Smurf-ness”).

Food uptake assays were undertaken as described in (21) using food containing 1% (w/v) erioglaucine disodium salt and 11.7 µM Zol as appropriate. Ten adult flies between 8 and 11 days old were placed in vials for 22 hours and allowed to feed ad libitum at 25°C. Flies were frozen and then dissociated in dH_2_O before centrifugation to pellet debris and extract color from internalized food. In addition, color present in fecal matter deposited on the walls of the vial was recovered by washing with dH_2_O. Color, and hence quantity of food consumed was calculated on the basis of optical density 630 nm measurements and reference to a standard curve of dye dilutions (Supplementary Figure 4A). Extracts from flies fed on undyed food were used as a blank. Ten groups of 10 flies were tested for each condition and gender and tested by 1-way analysis of variance (ANOVA). Final values are expressed as µg food per fly per day (correcting for the 22-hour sampling period).

### DNA Damage Assay

An overnight egg collection of *w*^*+*^*, GMR-Gal4,UAS-white*^*RNAi*^ adults outcrossed to OreR were allowed to develop for 96 hours at 25°C. These heterozygous third instar larvae were then irradiated with 200 Gy using a Torrex Cabinet X-ray system (Faxitron X-ray, Tucson, AZ) and adult flies that subsequently ecclosed were scored for the frequency of red *w*^*+*^ clones.

### *Drosophila* Midgut Analysis

Midguts from *Su(H)-lacZ; esg-Gal4,UAS-GFP/CyO* female adults were dissected in cold phosphate-buffered saline (PBS) and fixed in PBS+4% formaldehyde and blocked in PBS Tween (PBS, 0.1% Triton X-100, and 1% bovine serum albumin). Antibodies included Chicken anti-GFP 1:4000 (Abcam, Cambridge, United Kingdom) and anti-phospho-Histone H3 1:100 (New England Biolabs, Ipswich, MA) and secondary antibodies AlexaFluor 488 goat anti-chicken and AlexaFluor 594 goat anti-rabbit IgG (Life Technologies, Eugene, OR) were used. Stained guts were mounted with Fluoroshield with DAPI (Sigma Aldrich) and Grace Bio-Labs SecureSeal imaging spacers. Samples were imaged using Perkin Elmer Spinning Disk confocal microscope with a 40× objective. Images were then processed using Image J.

### Protein Extraction and Western Blot

Thirty flies of the appropriate genotype were snap-frozen in liquid N_2_ and crushed using an Eppendorf pestle in 200 µL ice-old lysis buffer (50 mM Tris–HCl pH 7.4, 250 mM NaCl, 5 mM ethylenediamine tetraacetic acid (EDTA), and 0.003% Triton X-100) with protease inhibitor (cOmplete Mini, EDTA-free, ROCHE, Sussex, United Kingdom). After 30-minute incubation at 4°C extracts were centrifuged at 13 000 rpm, 4°C for 30 minutes and supernatants stored at −20°C.

Proteins (40–60 µg) were electrophoresed through 4%–15% Mini-PROTEAN TGX Precast Protein Gels (Bio-Rad, Hertfordshire, United Kingdom) and transferred onto polyvinylidene difluoride membrane (Immobilon-P Membrane, Millipore, Hertfordshire, United Kingdom) using standard techniques. Primary antibodies used were anti-pAKT 1:500, anti-AKT 1:500, anti-tubulin 1:1000 (Cell Signaling Technology, Denver, Massachusetts). Horse radish peroxidase–conjugated secondary antibodies were used at 1:10 000 and visualized using ECL (GE Healthcare, Buckinghamshire, United Kingdom), high-performance chemiluminescence film (Amersham Hyperfilm ECL, GE Healthcare), and an Optimax 2010 X-ray film processor (PROTEC, Oberstenfeld, Germany).

### Statistical Analysis

Statistical analysis was performed on GraphPad Prism 7. For single comparisons data were analyzed using nonparametric unpaired Student’s *t* test. For multiple comparisons, ordinary 1-way ANOVA was used followed by Sidak’s multiple comparison post hoc tests. For the analysis of rapid iterative negative geotaxis assay 2-way ANOVA was used followed by Dunnet’s multiple comparison test to determine the effect of dose of the treatment at the different ages. Survival data were analyzed using Log-rank (Mantel–Cox) test to identify significant differences in organism survival between treatment groups. For the analysis of the Smurf assay chi-square test was used. All data are expressed as mean ± *SD*. A difference was stated to be statistically significant if the *p* value was <.05 (*p < .05; **p < .01; ***p < .001; ****p < .0001).

## Results

### Zol Increases Life Span in *Drosophila*

**Figure 1.**
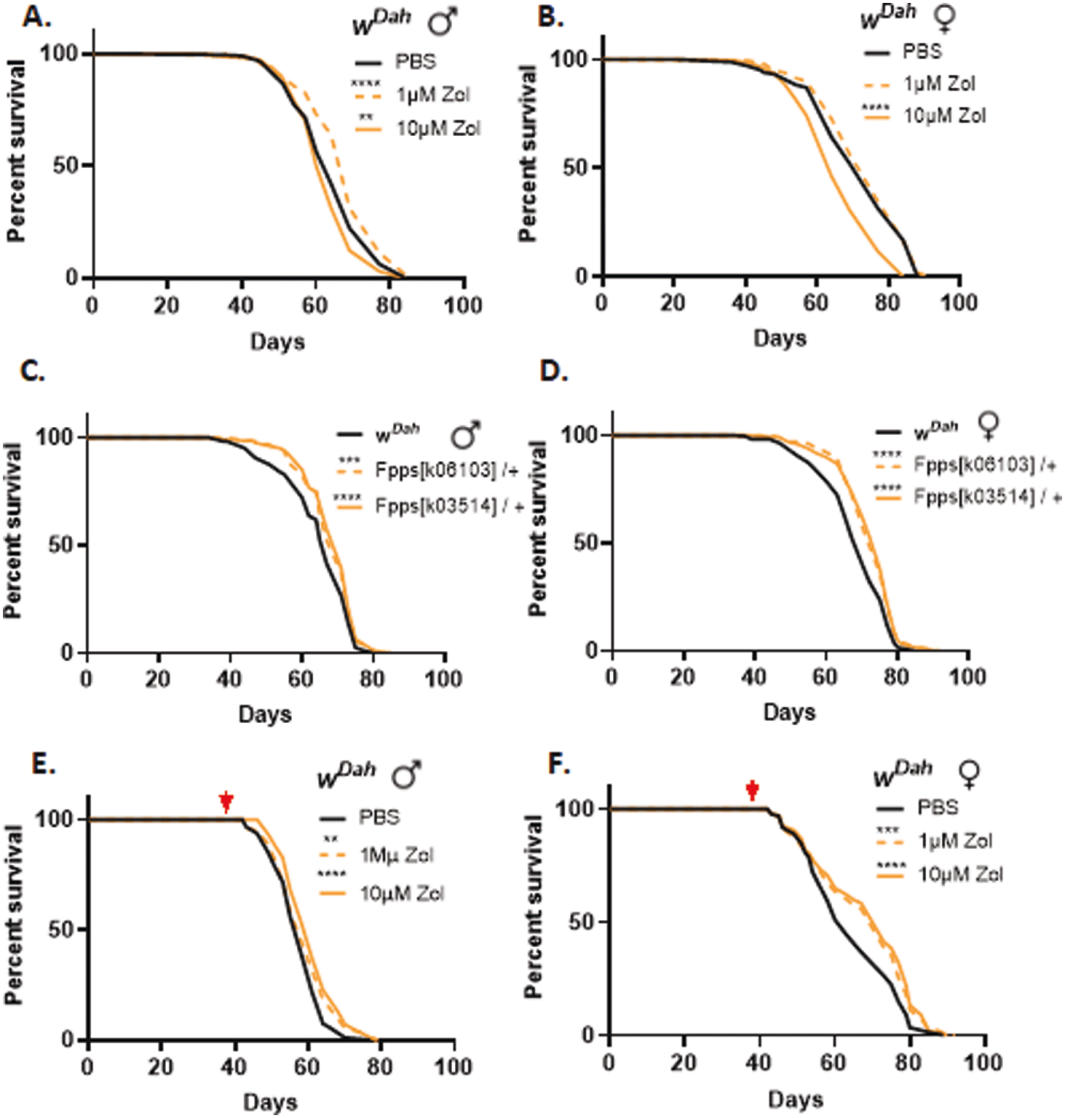
Administration of Zol affects life span of flies. Percentage survival of (**A**) male and (**B**) female *w*^*Dah*^ flies fed with food in presence or absence of Zol (1 or 10 μM) throughout their lives; percentage survival of (**C**) male and (**D**) female heterozygote FPPS mutant flies and *w*^*Dah*^ fed with standard *Drosophila* food; percentage survival of (**E**) male and (**F**) female *w*^*Dah*^ flies fed with food in presence or absence of Zol (1 or 10 μM) from 40 days of age. Average of 3 experiments with 100 flies/group/experiment. Log-rank (Mantel–Cox) test in Graphpad Prism used to statistically analyze the survival curves, **p ≤ .01, ***p ≤ .001, ****p ≤ .0001. All experiments were repeated 3 times.

To determine whether Zol might have beneficial effects on the life span of *Drosophila*, both male and female *w*^*Dah*^ flies were maintained on standard fly food supplemented with 1 or 10 μM Zol from Day 4 of adult life onwards (Figure 1A and B; Supplementary Figure 1). In multiple experimental replicates, the survival of males fed with 1 μM Zol was significantly increased compared to the vehicle-treated group (Figure 1A; Supplementary Figure 1). By contrast, females showed no significant beneficial effect when treated with Zol at 1 μM throughout their lives and 10 μM of Zol had adverse effects on female survival (Figure 1B).

As Zol modulates the mevalonate pathway through inhibition of FPPS, we next tested the life span of 2 heterozygous FPPS mutants (*Fpps*^*k06103*^*/+* and *Fpps*^*k03514*^*/+*)—2 independently generated homozygous-lethal loss-of-function alleles containing transposon insertions within the FPPS gene (15). Following at least 7 generations of outcrossing into the *w*^*Dah*^ genetic background, both mutant strains showed a statistically significant increase in overall survival in males and females compared to *w*^*Dah*^ controls (Figure 1C and D; Supplementary Figure 2) suggesting that reduced FPPS activity mediates an extension in life span.

To limit potential side effects due to long-term drug treatment, we next assessed the effects of Zol on life span when starting administration at Day 40 of adult life (middle age) (red arrow in Figure 1E and F; Supplementary Figure 3). This late treatment led to a significant increase in survivorship in both males and females for both 1 and 10 μM Zol compared to controls (Figure 1E and F; Supplementary Figure 3). This life-span extension was significant for both males and females with 1 μM Zol producing average median life-span increases of 1.79% (±0.10%) and 11.53% (±2.10%) (*N* = 3) for male and females, respectively, while with 10 μM Zol, survival was increased in males by 4.67% (±1.03%) and females by 16.51% (±4.94%) (*N* = 3). Finally, to verify that these results were not influenced by calorific restriction caused by unpalatable food we exposed the flies to drug-laced food containing a nontoxic food colorant via which the volume of food both present within flies and excreted can be measured. No significant difference in food uptake was observed in presence of Zol in either males or females (Supplementary Figure 4*n* = 10/trial, 10 trials per condition), excluding the possibility that the life-extending effects were the result of caloric restriction.

### Zol Increases Health Span in *Drosophila*

**Figure 2.**
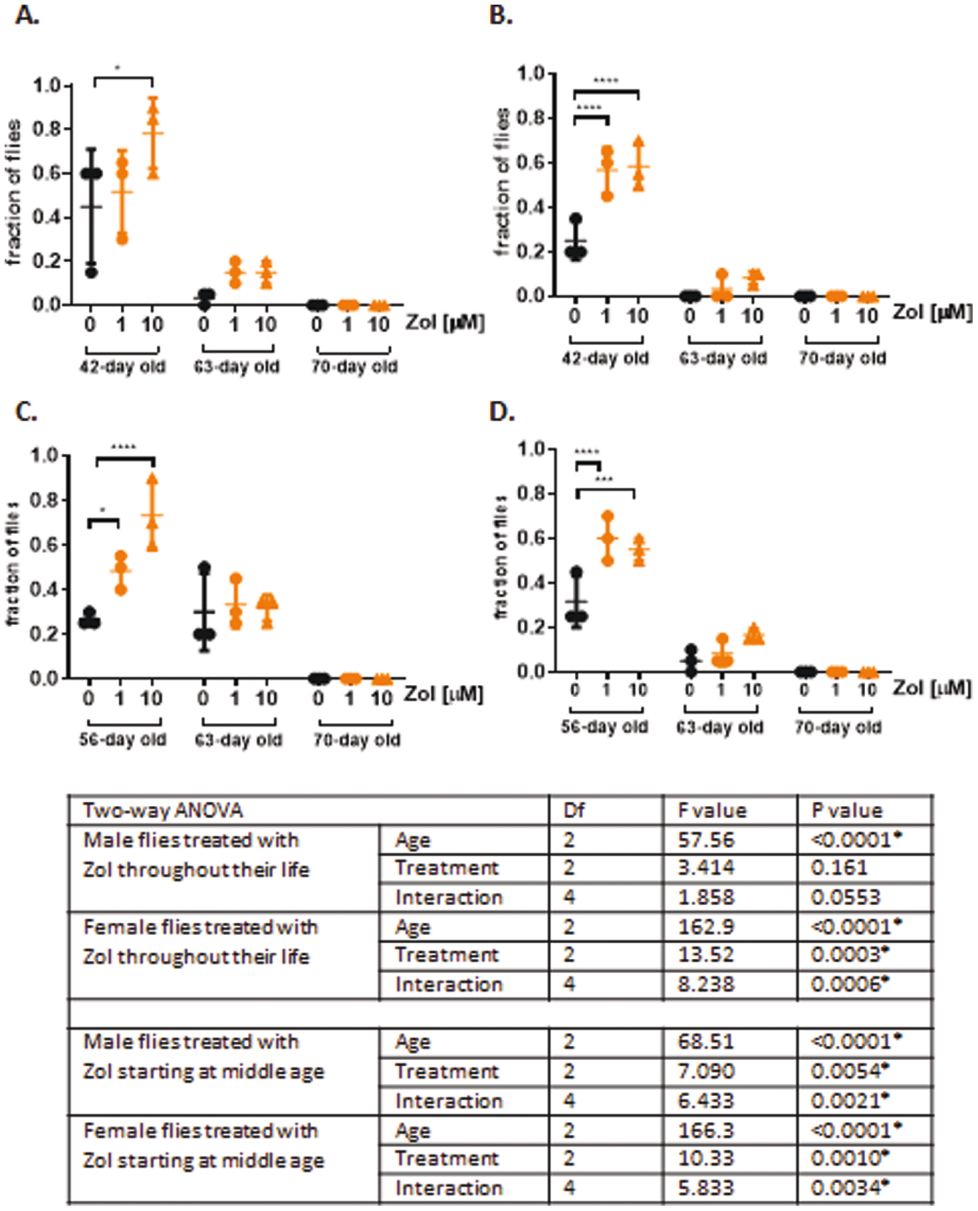
Flies fed with Zol shows a significant increase in climbing ability at middle age. Lifelong treatment with Zol: (**A**) Percentage of male flies climbed above 10 cm (high climbers) within 15 seconds. (**B**) Percentage of female flies climbed above 10 cm (high climbers) within 15 seconds. Flies treated with Zol from midlife: (**C**) Percentage of male flies climbed above 10 cm (high climbers) within 15 seconds. (**D**) Percentage of female flies climbed above 10 cm (high climbers) within 15 seconds. Data were analyzed by 2-way ANOVA and Dunnet’s multiple comparison test. *p ≤ .05, **p ≤ .01, ***p ≤ .001, ****p ≤ .0001.

To determine whether Zol improved signs of health span we next tested its effects in *Drosophila* by assessing their ability to climb and by the presence of signs of intestinal epithelial dysplasia and intestinal permeability. Displaying strong negative geotactic responses, climbing activity is a widely used assay of *Drosophila* health and activity (22) and was assessed using the rapid iterative negative geotaxis assay (Figure 2). We classed as “high climbers” flies able to climb over 10 cm in 15 seconds. As expected, the percentage of high climbers decreased significantly with age in both males (Figure 2A and C) and females (Figure 2B and D). However, a significant improvement in climbing ability was observed in both males and females receiving Zol from Day 4 of adult life (Figure 2A and B) or starting at 40 days of life (Figure 2C and D) when assessed at 42 and 56 days of age, respectively. Two-way ANOVA analysis showed that, despite a significant interaction between age and treatment (Figure 2 for details of statistical analysis), no significant effect of Zol was observed at a later time point in any of the conditions analyzed by post hoc test. These data show there is an overall improvement in climbing ability at middle age, which is independent of the effects on life span.

**Figure 3.**
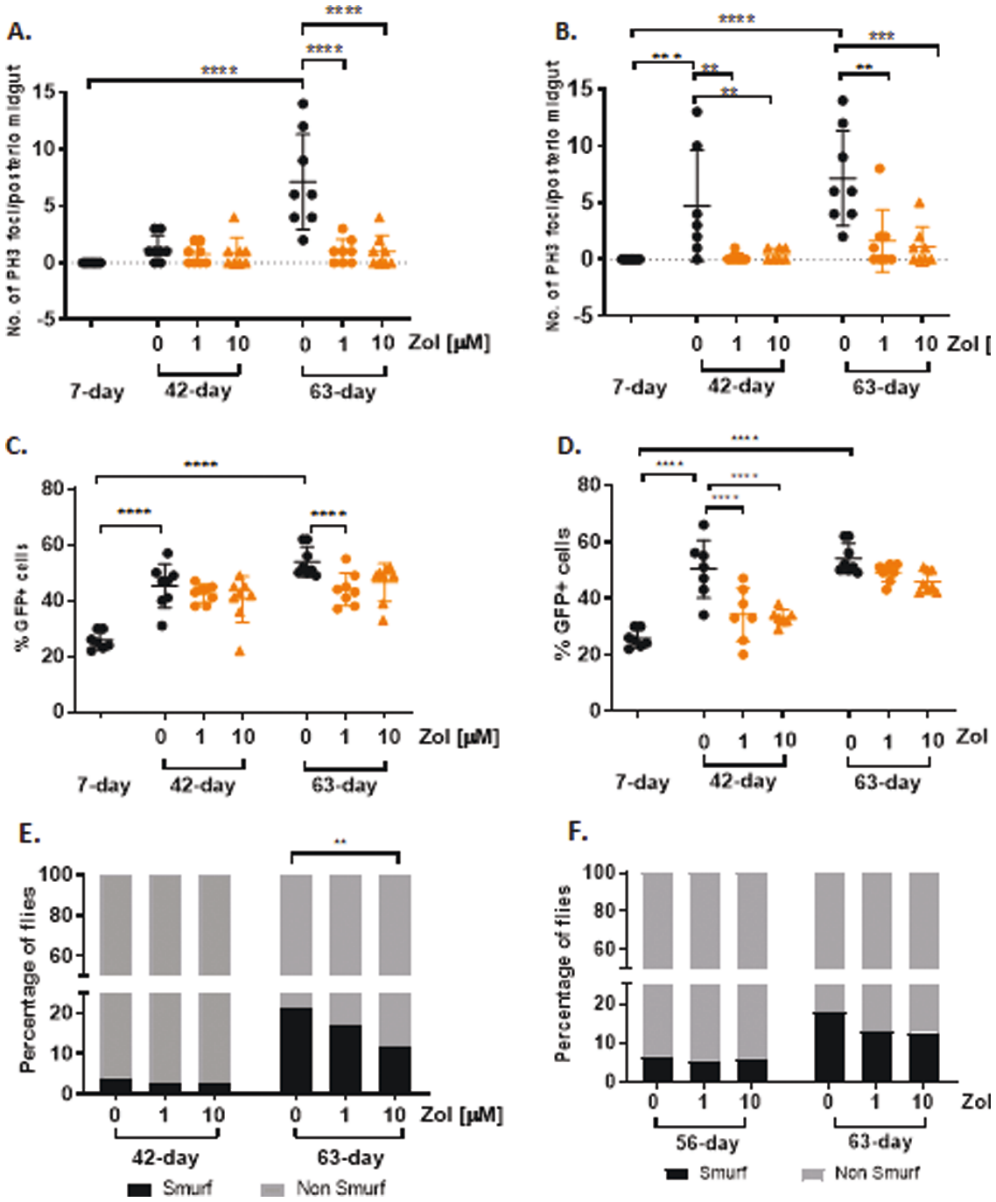
Treatment with Zol reduces epithelial dysplasia and intestinal permeability in *Drosophila*. (**A, B**) Quantification of pH3+ cell in the intestine of *Drosophila*. Flies were treated with or without 1 or 10 μM of Zol from 4 days of age (**A**) or 40 days of age (**B**). At least 7 midguts were assessed per condition and per time point. (**C, D**) Quantification of %GFP+ cells (normalized to DAPI) in the intestine of flies treated with or without 1 or 10 μM of Zol from 4 days of age (**C**) or 40 days of age (**D**). (**E, F**) Percentage of flies Smurf positive following treatment with or without 1 or 10 μM of Zol starting from 4 days of age (**E**) or 40 days of age (**F**) (*n* > 65 per condition per time point). Data were analyzed with 1-way ANOVA and Sidak’s post hoc test (number of PH3+ cells and %GFP+ cells) and chi-square test for the analysis of the Smurf assay. **p < .01, ***p < .001, ****p < .0001.

As flies age, intestinal barrier dysfunction and epithelial dysplasia develop in female flies, a development associated with increased mortality and a development considered to be a good marker of health span (23). To determine the effects of Zol on epithelial dysplasia we examined intestinal morphology at Day 7, 42, and 63 of adult life following treatment with vehicle or Zol starting at either Day 4 or 40 of adult life. Using *Su(H)-lacZ; esg-Gal4,UAS-GFP* reporters we labeled intestinal stem cells and enteroblasts (progenitor cells) on the basis of GFP expression driven by the *escargot* promoter—a marker associated with stemness. Epithelial dysplasia in the *Drosophila* gut is characterized by hyperproliferation, as identified by the G2/M marker phospho-Histone3 (pH3) (24) and misdifferentiation of intestinal stem cells (25). As expected, we observed an increase in intestinal stem-cell proliferation with age in vehicle-treated flies as shown by the significant increase in the number of GFP+/pH3+ cells with age (Figure 3A and B) and an increase in the overall proportion of GFP+ cells (Figure 3C and D). Both parameters are significantly decreased in female flies treated with Zol (Figure 3A–D).

One of the key roles played by the intestinal epithelia is to provide an impermeable barrier to the exterior environment in which damaged or dying epithelial cells are replaced with new cells generated by the intestinal stem cells. A loss of barrier function in the intestinal epithelium has been reported with age in both flies and humans. To assess intestinal integrity we performed the “Smurf assay” (26) in which blue coloring added to regular food is able to cross a compromised epithelium and stain the entire fly blue. Consistent with previous findings, the percentage of flies presenting the “Smurf” phenotype was increased with age and is attenuated by treatment with Zol under the all-life feeding regime (Figure 3E and F).

Taken together, these data suggest that treatment with Zol throughout adult life reduces intestinal dysplasia and permeability in *Drosophila*.

### Zol Increases Life Span of Flies Exposed to Oxidative Stress Through FOXO

**Figure 4.**
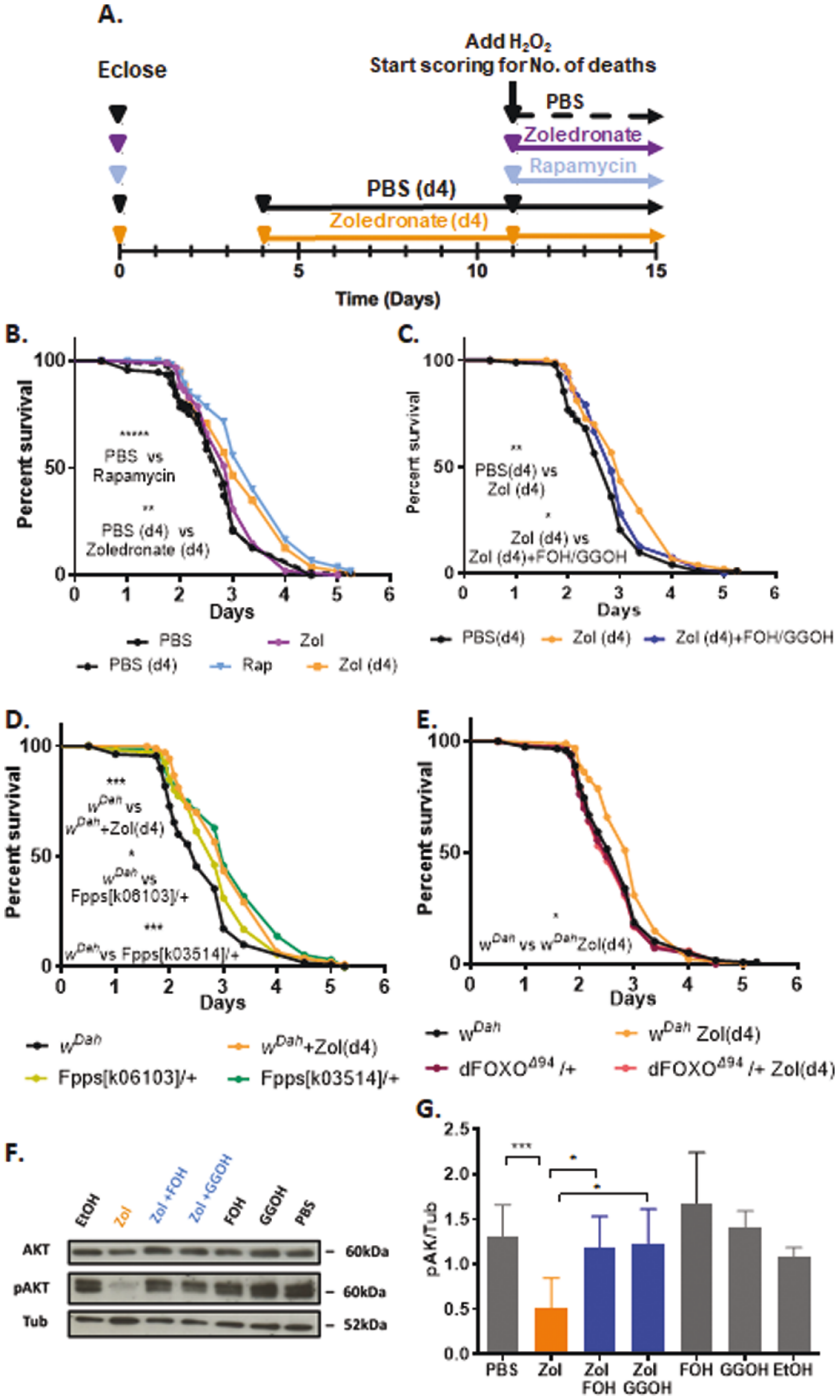
Zol increases life span of flies under oxidative stress through FOXO. (**A**) A schematic representation of the experimental design. (**B**) Survivorship of female *w*^*Dah*^ flies fed on food containing 5% H_2_O_2_. The flies were treated with 5% H_2_O_2_ in combination with PBS (vehicle) or 10 μM Zol or 200 μM rapamycin (Rap) both given at the same time than H_2_O_2_. Two groups of flies were pretreated for 7 days with 10 μM Zol (d4) or PBS (d4) as vehicle before addition of H_2_O_2_. (**C**) Survivorship of female *w*^*Dah*^ flies fed with 5% H_2_O_2_. The flies were pretreated with PBS+EtOH (vehicles), 10 μM Zol, or 10 μM Zol in combination with 330 μM FOH and 330 μM GGOH. (**D**) Survivorship of heterozygote female FPPS mutants: *Fpps*^*k06103*^*/+* and *Fpps*^*k03514*^*/+* fed with 5% H_2_O_2_. *w*^*Dah*^ flies either pretreated with PBS or 10 μM Zol were used as control. (**E**) Survivorship of female dFOXO^Δ94^/+ flies treated with 5% H_2_O_2_. *w*^*Dah*^ flies either pretreated with 10 μM Zol or PBS (vehicle) were used as control. For all survival tests, 100 flies were used in each treatment group per test, experiments were repeated 3 times with different cohorts of flies. (**F**) A representative example of AKT and pAKT expression in whole flies fed with Zol (100 μM) for 10 days, in presence or absence of FOH (330 μM), GGOH (330 μM). PBS and ethanol (EtOH) were used as vehicle control. (**G**) Quantification of expression level of pAKT normalized to tubulin in presence or absence of Zol (100 μM), FOH (33 μM), and GGOH (33 μM) for 10 days analyzed by ImageJ (*n* = 3). *p ≤ .05, ** ***p ≤ .001.

To elucidate the mechanism of action mediating the beneficial effects of Zol and considering the role of GTPases in longevity we hypothesized that resistance to oxidative stress was partly responsible for the increased survivorship. Therefore, flies were challenged with food containing 5% H_2_O_2_, a treatment that reduces absolute life span and acts as a source of oxidative stress. Groups of adult, 11-day-old female *w*^*Dah*^ flies were subjected to 5% H_2_O_2_ at the same time as either Zol or rapamycin used as a positive control (27). We also tested another group of flies pretreated with Zol for 7 days before exposure to 5% H_2_O_2_ (Figure 4A). While the survival of flies treated with rapamycin increased significantly compared to vehicle control, Zol treatment did not improve survival when administered at the same time as the 5% H_2_O_2_ (Figure 4B). By contrast, survival in response to H_2_O_2_ was significantly increased following pretreatment with Zol for 7 days (Figure 4B). To determine whether the increase was due to inhibition of FPPS, flies pretreated with Zol were also treated with the downstream metabolites of the mevalonate pathway, FOH and GGOH, which bypass the block in FPPS inhibition. As expected, the survival advantage provided by Zol pretreatment was abrogated in the presence of FOH and GGOH demonstrating that the extension in survival is due to inhibition of the mevalonate pathway (Figure 4C). To further support the hypothesis that inhibition of FPPS is responsible for the resistance to oxidative damage, *Fpps*^*k06103*^*/+* and *Fpps*^*k03514*^*/+* heterozygotes outcrossed into a *w*^*Dah*^ genetic background were also subjected to 5% H_2_O_2_ and their survival assessed. In these flies, survival was also significantly increased compared to *w*^*Dah*^ controls following exposure to H_2_O_2_-induced oxidative stress (Figure 4D). Taken together these data suggest that the Zol confers resistance to oxidative stress and demonstrates that these effects are mediated by inhibition of the FPPS enzyme in the mevalonate pathway.

To determine the mechanism of action downstream of the mevalonate pathway, we hypothesized that Zol may be affecting the activity of dFOXO, a factor regulating oxidative stress signals. To determine whether Zol protects *Drosophila* from oxidative damage through dFOXO, heterozygous *dFOXO*^*Δ94*^*/+* loss of function mutations (17) and *w*^*Dah*^ controls were pretreated with either Zol at the highest dose of 10 µM or vehicle control-containing food for 7 days before exposure to food containing 5% H_2_O_2_. While *w*^*Dah*^ pretreated with Zol showed a significant increase in life span, *dFOXO*^*Δ94*^*/+* flies treated in the same way did not show any beneficial effect (Figure 4E), suggesting dFOXO is required for Zol action on survival.

Given that pAKT is a known regulator of FOXO (28) we next determined levels of pAKT, following treatment with Zol and observed a significant decrease (Figure 4F; Supplementary Figure 5). Furthermore, these effects were reversed by the addition of FOH and GGOH (Figure 4F and G), suggesting that inhibition of pAKT is dependent on the mevalonate pathway.

Taken together these data suggest that Zol confers resistance to oxidative damage via inhibition of the mevalonate pathway through a mechanism that requires dFOXO for its activity.

### Zol Enhances DNA Damage Repair in *Drosophila* Upon Irradiation

**Figure 5.**
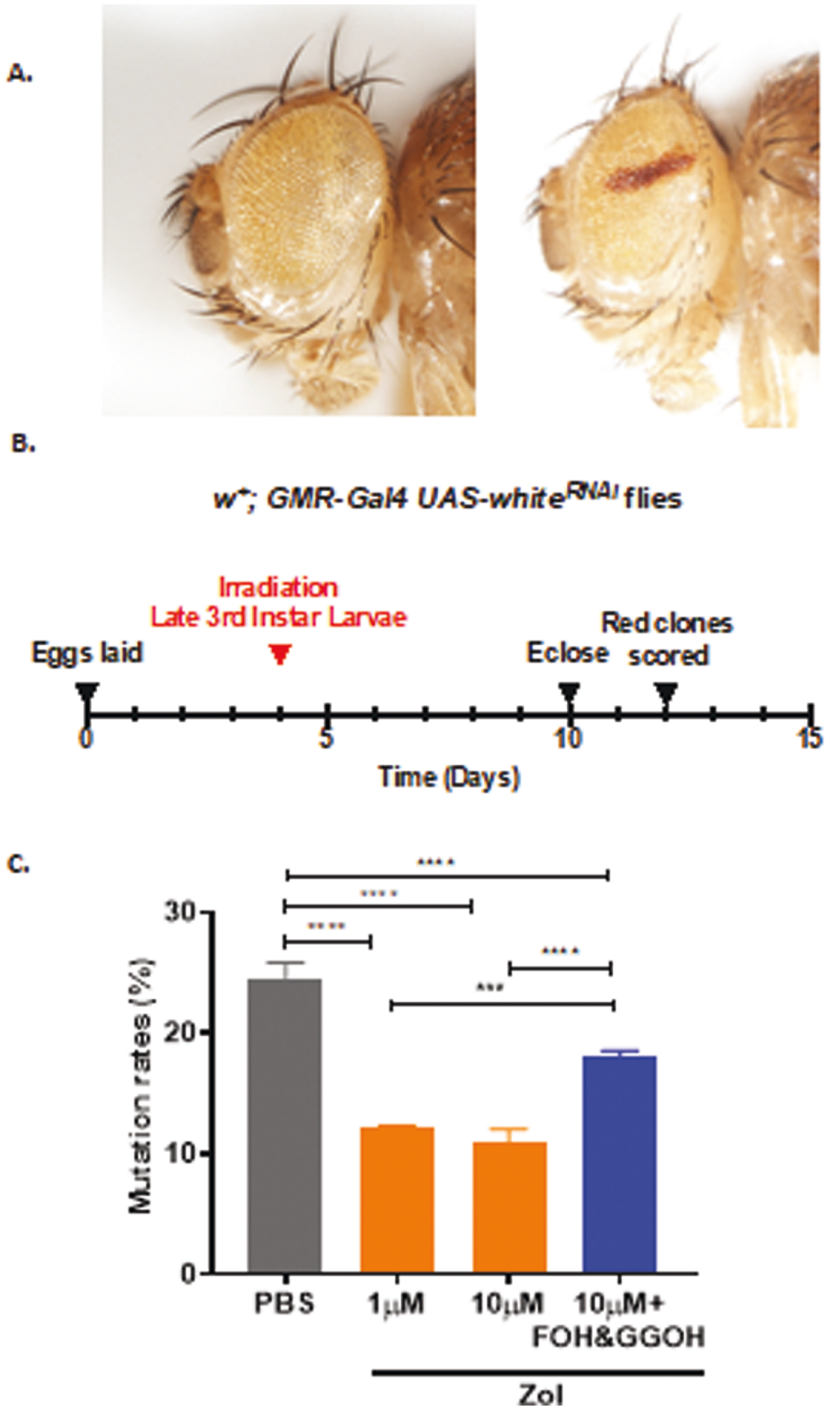
Zol enhances DNA damage repair in *Drosophila* upon irradiation. (**A**) A representative image of an eye from a *w*^*+*^*; GMR-Gal4,UAS-white*^*RNAi*^ fly (left) and one containing a clone of cells in the eye in which DNA damage has led to the production of red pigment (right). (**B**) Time line of experiment. (**C**) Percentage of eyes with red colonies upon 200 Gy irradiation in homozygous *w+; GMR-Gal4,UAS-white*^*RNAi*^ flies when treated with PBS (control), 1 μM Zol, 10 μM Zol, or 10 μM Zol with 330 μM FOH and GGOH. Data analyzed by 1-way ANOVA and Sidak post hoc test. ***p < .001, ****p < .0001.

DNA damage and the resulting mutations induced by ionizing radiation are at least partly caused by increased oxidative stress (29). We therefore wanted to determine whether the ability of Zol to protect against H_2_O_2_-induced oxidative stress would also reduce the frequency of DNA damage induced by X-ray irradiation. In order to visualize DNA damage in an in vivo environment, we developed an assay in which endogenously expressed mRNA from the wild-type *white* locus is knocked down by an in vivo RNAi hairpin-loop expressed within the cells of the future eye. Where knockdown is successful, *white* mRNA is destroyed and very little pigment is produced, resulting in pale yellow eye pigmentation. Where the DNA encoding components of the *GMR-Gal4,UAS-white*^*RNAi*^ expression system are mutated, *white* mRNA is not destroyed and wild-type levels of red pigment are produced to give a readily recognizable red eye clone (Figure 5A) in adult *Drosophila*. In order to assess DNA damage levels using this reporter, larvae heterozygous for the *GMR-Gal4,UAS-white*^*RNAi*^ reporter were raised on food containing either carrier controls, 1 μM Zol, 10 μM Zol, or a combination of 10 μM Zol and 33 μM FOH and 33 μM GGOH. Larvae were irradiated 96 hours after hatching using 1 dose of 200 Gy of X-ray irradiation (Figure 5C). Strikingly, *GMR-Gal4,UAS-white*^*RNAi*^*/+* flies contained significantly lower frequency of red-marked mutated cells when grown on 1 or 10 μM Zol-containing food compared to controls. However, when *Drosophila* were treated with 10 μM Zol in combination with FOH and GGOH, a partial reversal of this effect was observed (Figure 5C). These data suggest that Zol is also able to protect individuals from the accumulation of mutations, via mechanism(s) that, at least in part, depend on the mevalonate pathway.

## Discussion

In this study we show that Zol has properties of a geroprotector, an activity mediated by its inhibition of FPPS. We show that Zol extends the life span and health span of *Drosophila* in absence of mineralized bone-like structures and demonstrate that it confers resistance to oxidative damage via the inhibition of FPPS in the mevalonate pathway. The median extension of life span by Zol is in the same range of other geroprotectors including rapamycin, with Zol extending the median life span of females by 14%–18% when given from middle age. Rapamycin, one of the most studied geroprotectors, which positively affects the immune system in older patients by boosting their ability to improve flu vaccine responses (30) and also improves several health-span parameters in mice (31), increases life span by 9%–14% in female mice when fed late in life (32). A similar level of extension was shown in other studies testing rapamycin in *Drosophila* (33).

Our results suggest that Zol requires dFOXO for its action and is associated with reduction in the levels of pAKT. These in vivo findings are in line with our previous work in human mesenchymal stem cells where we have shown that Zol was able to reduce the accumulation of DNA damage caused by cellular aging or irradiation via a mechanism requiring FOXO3a, as well as reducing the accumulation of senescent markers p16 and p21 (34). In addition, Zol was able to increase the translocation of pFOXO3a to the nucleus in human mesenchymal stem cells and this was reversed by the addition of FOH and GGOH, suggesting an action on the activation of FOXO3a (34). Molecules such as Rheb and Ras are small GTPases prenylated via the mevalonate pathway and upstream of TORC1 and TORC2 and therefore are likely candidates to mediate the regulation of pAKT and FOXO by Zol (35,36) (a schematic representation of the hypothetical pathway is in Supplementary Figure 6). FOXO is a key player in aging and has been shown to regulate several of its hallmarks including DNA damage, senescence, changes in mitochondrial function, and mutation rates (37). While *Drosophila* have a single FOXO gene (dFOXO), the human genome is more complex and encodes 4 different FOXO proteins, with polymorphisms in FOXO3a having been associated with exceptional longevity (38,39). In addition, FOXO3a activity has been shown to reduce the effects of reactive oxygen species production in multiple ways. For example, its expression improves the fidelity of DNA damage repair by arresting the cell cycle to allow the repair of damaged DNA (40,41). In addition, FOXO3a activation results in the repression of a large number of nuclear-encoded genes with mitochondrial function (42). As most intrinsic reactive oxygen species are produced by the respiratory complexes located in the inner mitochondrial membrane, these changes in mitochondrial activity may directly influence the levels of reactive oxygen species production in vivo. More work is required to understand in detail which of these mechanisms and molecular pathways Zol modulates via FOXO. In addition, it will be important to determine which of the many small GTPases modulated by the mevalonate pathway are responsible for FOXO activity. A detailed analysis of each individual tissue in a mammalian system is also required as there are important differences in response to oxidative stress not only among tissues but even within regions of the same tissues (43).

A similar molecular mechanism mediating extension of life span has been described following administration of statins in *Caenorhabditis elegans*. Statins inhibit HMG-Co-A reductase in the mevalonate pathway and extend life span via DAF16/FOXO3a (44). However, statins are administered daily as opposed to Zol which is given once a year in patients affected by osteoporosis. This can be an advantage in terms of cost-effectiveness, especially when considering preventive interventions.

One observation we made is that the effects of Zol varied depending on time of administration, dose, and sex differences that may be partly due to differences in drug uptake by males and females. In females, egg production requires higher levels of nutritional input than required by males, resulting potentially in increased food consumption and therefore increased drug uptake with consequent signs of toxicity (45). By contrast, in the FPPS mutants, where the action of the enzyme is disrupted independently of drug uptake, similar life-span extension is observed in both sexes suggesting that the effect of mevalonate pathway inhibition on life span is unlikely to be sexually dimorphic. However, further work is required to understand this aspect of the work.

In addition to differences in life-span extension, it is intriguing to note that indicators of improved health such as climbing ability and intestinal dysplasia occur even at high doses of Zol that reduce absolute life span (compare Figure 1B 10 µM with Figure 3A, 63-day 10 µM), demonstrating that drug treatment is able to increase health span independently of absolute life span. This observation is in line with the finding of others which shows that health span and life span are not necessarily related (46). For example, Nicotinamide has recently been shown to improve aspects of health span but not life span (47). This disconnect between health span and life span may indicate that organisms can cope with the accumulation of a certain number of defects which are sufficient to negatively affect health span but which do not lead directly to death. Being able to reduce these health span-associated deficits is of particular interest from a translational perspective as they contribute to morbidity and poor health and are responsible for significant health care costs.

Another notable aspect of Zol treatment in *Drosophila* is the improvement in life-span and health-span measures following treatment that begins only in middle age (Figure 1E and F). It is unclear why the effects are more prominent when the treatment starts at middle age, particularly in females. The treatment profile observed in these flies mirrors that of women affected by osteoporosis who also generally begin Zol treatment postmenopausally. In both *Drosophila* and humans, the effect of Zol on survival is only detectable sometime after initial treatment (48). On average, it takes 16 months before an improvement in survival is observed in postmenopausal women (48) and approximately 14 days in *Drosophila* (Figure 1F). The reasons for this delayed phenotypic response are unclear but may reflect an ability by Zol to improve biological processes only when they are mildly dysregulated. However, when those mechanisms are either working at healthy levels or when their dysregulation exceeds a compensatory threshold, then Zol treatment is either not needed or no longer sufficient to maintain function. Consistent with this model, Zol did not have any effect on climbing activity in geriatric flies at 70 days of age—when no activity was detected in any of the populations tested. It is possible that a similar process may also explain why a Zol-mediated extension of life span was not observed in *Zmpste24*^*−/−*^ mice. In this model of Hutchinson–Gilford progeria syndrome Zol alone does not modify premature aging while combined treatment with both Zol and statins (which inhibit the same pathway) is able to extend life span (49).

While evidence collected to date suggests that the effects of Zol on life span and health span in humans may be limited, it should be noted that the effects observed follow treatment with just a single yearly dose. Nonetheless, retrospective analysis of patients taking Zol has not only shown a marked increase in survival but also a reduction in the frequency of death by pneumonia and cardiovascular events, suggesting broader effects unrelated to the musculoskeletal system (9). In addition, patients treated with Zol and admitted to hospital for intensive/critical care for a condition not related to osteoporosis showed reduced mortality rates relative to controls (5.2% vs 9.1%, respectively). Furthermore, the patients previously treated with Zol required 30% shorter in-patient care despite being older than the control group and having higher comorbidity index (10).

Older people with frailty and multimorbidity have a reduced ability to respond to adverse events and often lose independence following major health-related incidents. Such events have significant consequences for follow-up health and social care costs (50,51). Indeed, there has been an increase of 18% in the number of emergency admissions of older patients between 2010/2011 and 2014/2015 in the United Kingdom with those patients over the age of 65 now accounting for 62% of total bed days spent in hospital. Considering average cost for a patient to stay in an National Health Service ward is up to £400 per day, the financial and societal benefits of improving resilience in older people and reducing length of hospital stay are huge (51).

In conclusion we have shown that inhibition of FPPS by Zol modulates mechanisms of aging to extend life span and health span in vivo—an effect that is independent of its effects on bone. These findings are in line with the unexplained improved survival rates that have been reported recently for patients being treated with Zol—findings that highlight the substantial benefit Zol can potentially provide with only a single yearly treatment. Studies in mammalian models are now required to understand the effects of Zol on specific tissues, to define which of the FOXO-regulated mechanisms are at play and whether an infrequent administration of Zol is the best approach to elicit the strongest beneficial effects. Given that Zol is off-patent, available at low cost, and displays a well-understood safety profile featuring minimal side effects, we suggest that repurposing studies seeking to widen the use of Zol are likely to identify great potential for the improvement of health span and resilience in older people.

## Supplementary Material

glab172_suppl_Supplementary_Figures

## Data Availability

The data that support the findings of this study are available from the corresponding author upon reasonable request.
