# Supplementary material for: Zoledronate Extends Health Span and Survival via the Mevalonate Pathway in a FOXO-dependent Manner
Source: J Gerontol A Biol Sci Med Sci. 2021 Jun 17;77(8):1494–502. doi: 10.1093/gerona/glab172 (PMC9373971; doi:10.1093/gerona/glab172)
Supplement: glab172_suppl_Supplementary_Figures [file glab172_suppl_supplementary_figures.pdf]

eFig. 1

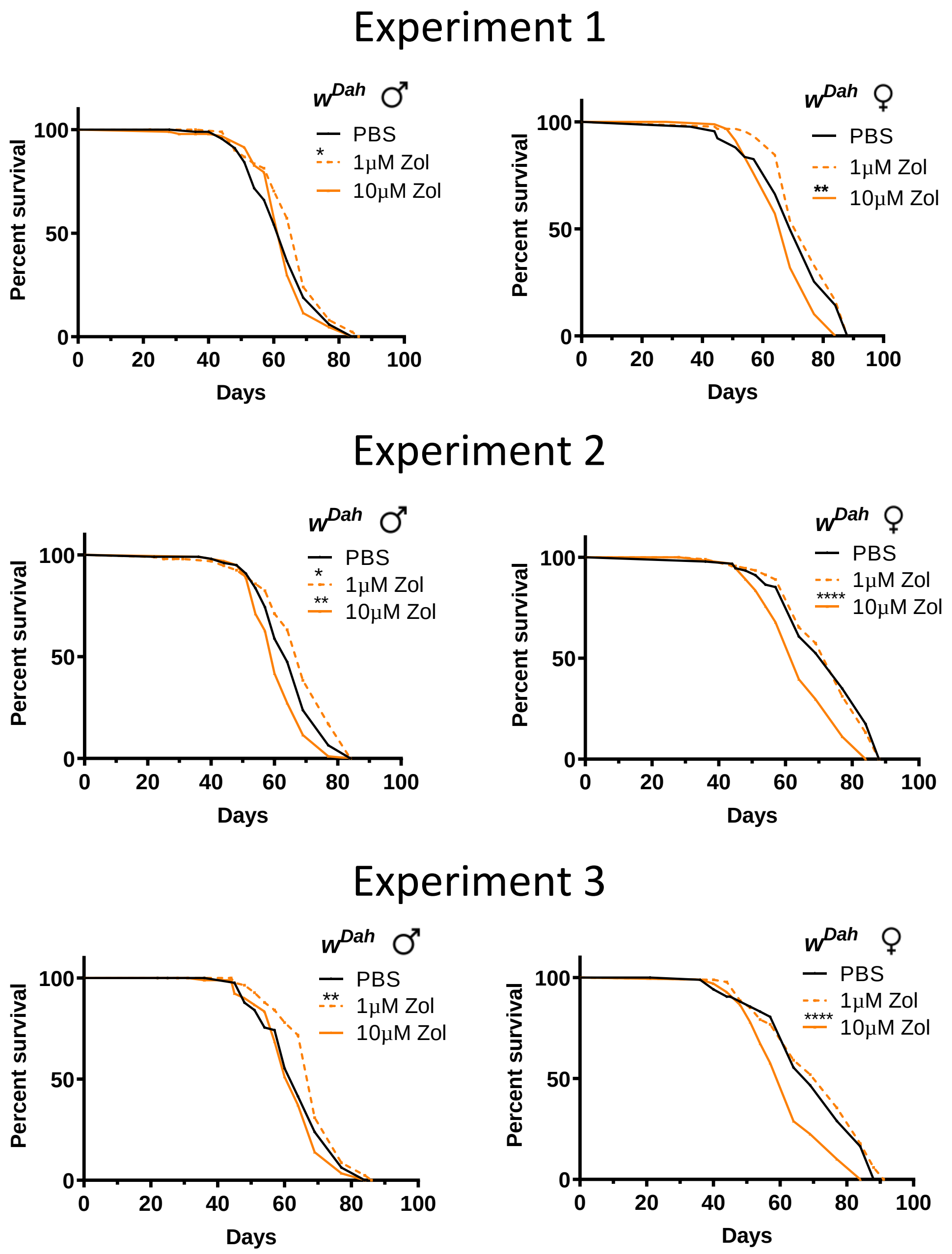

eFig. 1 Administration of Zol from day 4 affects lifespan of flies in males but not in females. Percentage survival of male (left column) and female (right column) w^{Dah} flies fed with food in presence or absence of Zol (1 or 10µM) throughout their lives. Representation of 3 replicate experiments containing 100 flies/group each. Data were analysed by Log-rank (Mantel-Cox) test in Graphpad Prism used to statistically analyse the survival curves, * $P \leq 0.05$, ** $P \leq 0.01$, **** $P \leq 0.0001$

eFig. 2

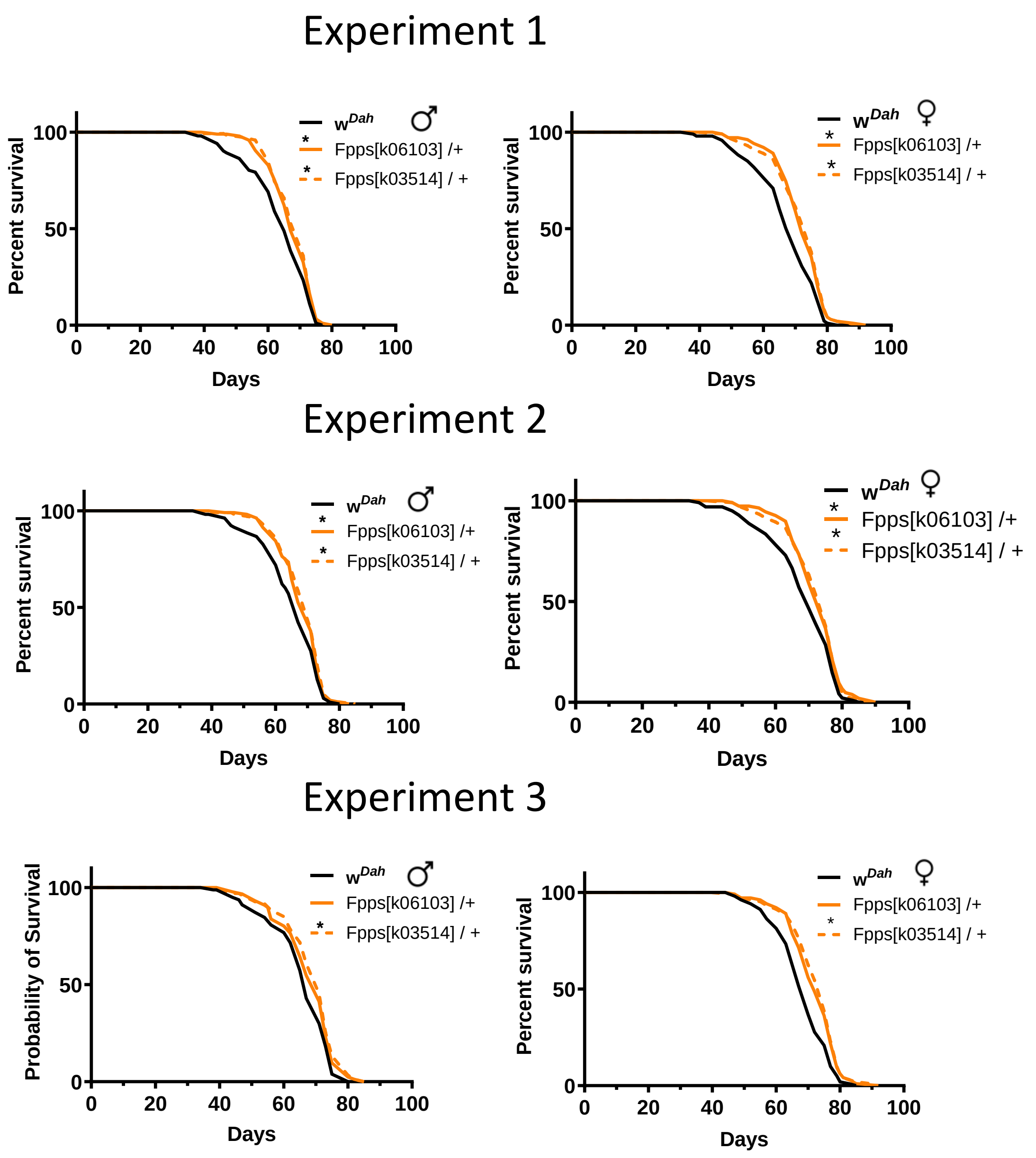

eFig. 2 W^{Dah} male and female heterozygote Farnesyl phyrophosphate synthase (FPPS) mutant flies showed an extension in lifespan. Percentage survival of male (left column) and female (right column) w^{Dah} FPPS flies. Representation of 3 replicate experiments containing 100 flies/group each. Data were analysed by Log-rank (Mantel-Cox) test in Graphpad Prism used to statistically analyse the survival curves, * $P \leq 0.05$, ** $P \leq 0.01$, **** $P \leq 0.0001$

eFig. 3

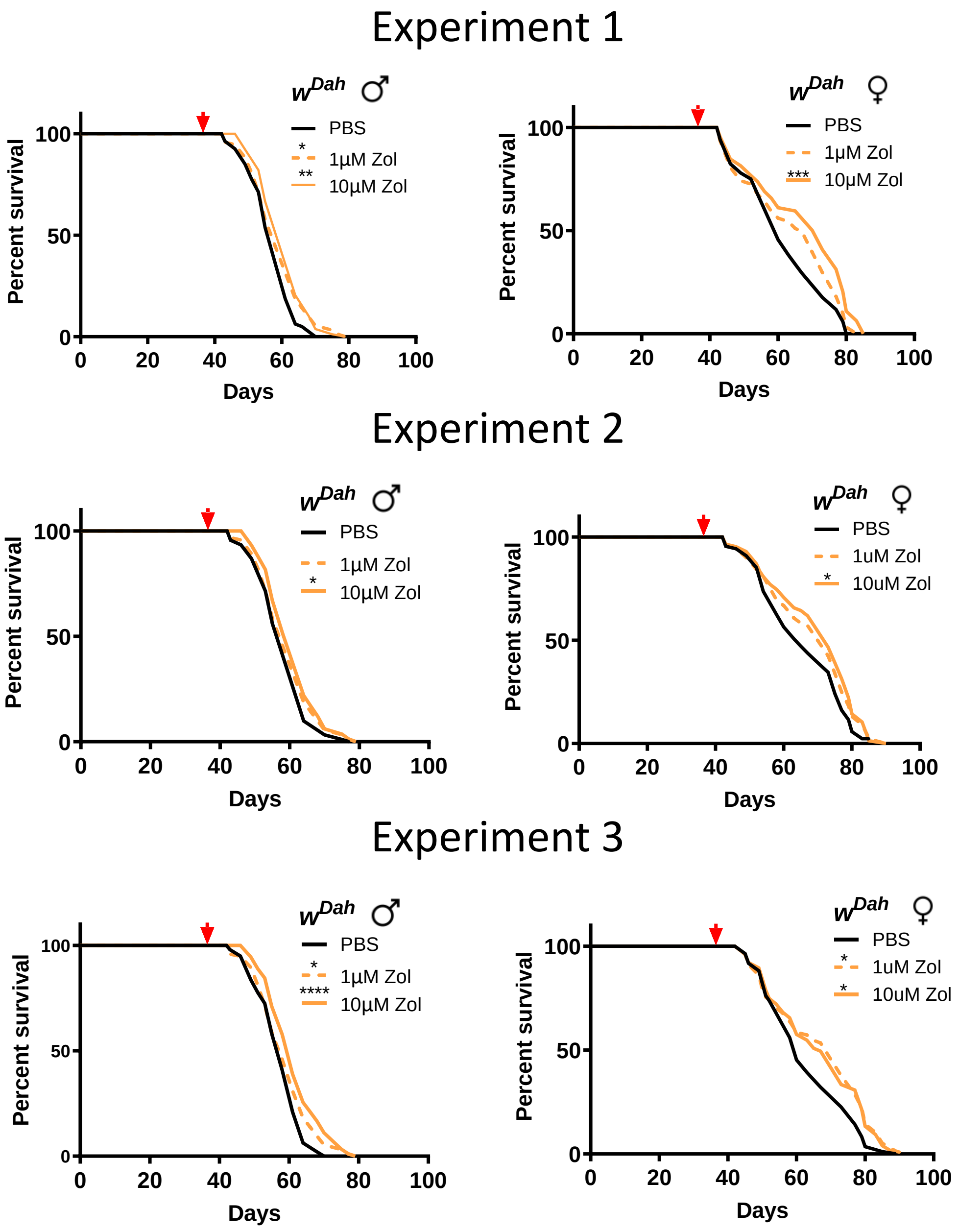

eFig. 3 Administration of Zol from day 42 (middle age) affects lifespan of flies in males and females. Percentage survival of male (left column) and female (right column) *w*^{Dah} flies fed with food in presence or absence of Zol (1 or 10μM) from day 42 of their life. Representation of 3 replicate experiments containing 100 flies/group each. Data were analysed by Log-rank (Mantel-Cox) test in Graphpad Prism used to statistically analyse the survival curves, * P ≤ 0.05, ** P ≤ 0.01, ***P,0.001, **** P ≤ 0.0001

eFig. 4

A.

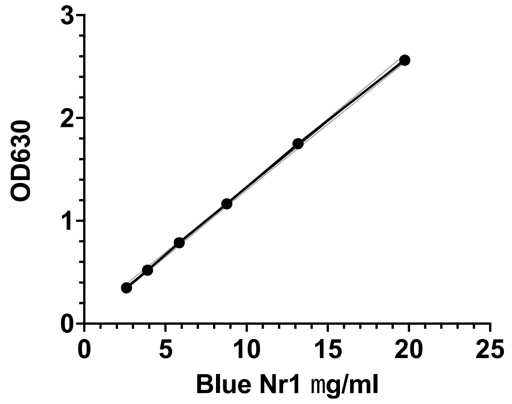

B.

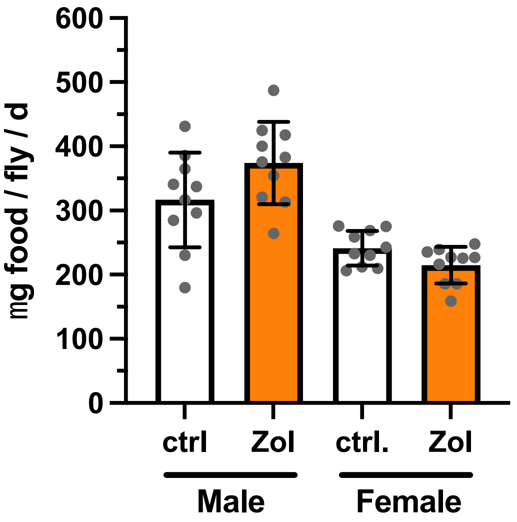

eFig. 4. Food intake level was not affect by the addition of Zol to the diet
(A) Standard curve of increasing concentrations of Blue Nr1 in distilled water and the correspondent OD 630 reading. (B) Quantification of total food consumed by 8-11d old Male and Female *Drosophila* eating either normal (ctrl, white) or Zol-containing (orange) food. Individual replicates (of 10 flies each) are show as grey dots and were analysed by one-way ANOVA and Dunnet's multiple comparisons test.

eFig. 5

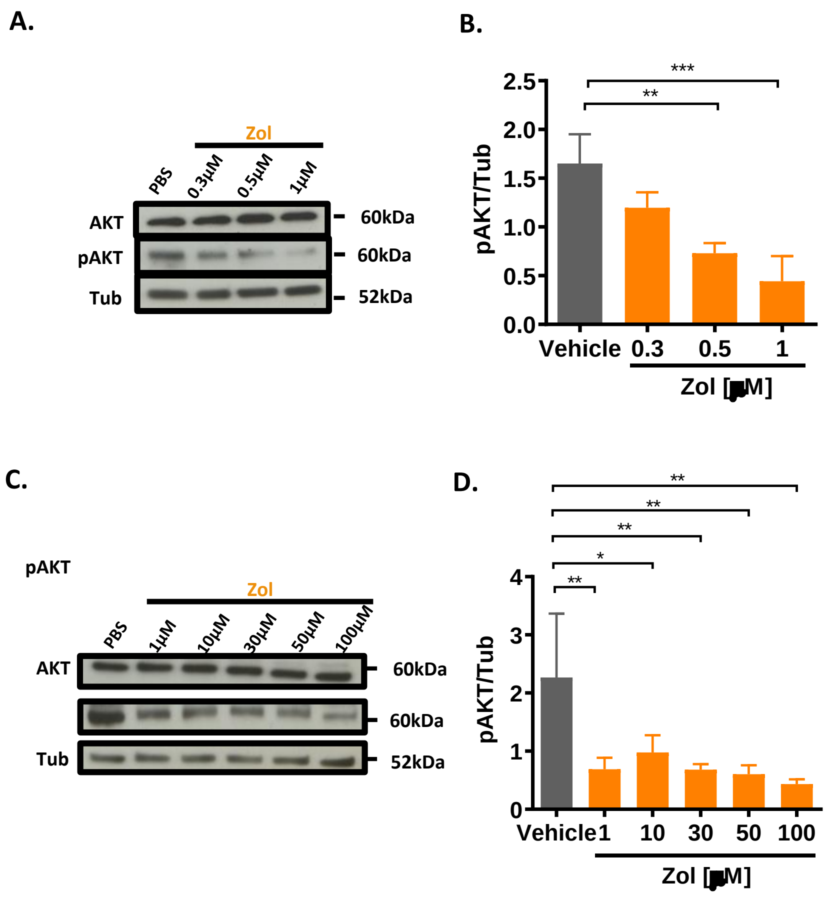

eFig. 5 Zol reduces pAKT level in Drosophila in a dose-dependant way. (A) A representative example of AKT and pAKT expression in whole flies fed on food containing 0.3- 1µM Zol for 10 days. Group of flies were also fed with food containing PBS only (vehicle) for control. (B) Quantification of expression level of pAKT normalised to tubulin (Tub) in presence or absence of Zol (0.3-1µM) in Drosophila food for 10 days analysed by imageJ (n=3). (C) A representative example of AKT and pAKT expression in whole flies fed on food containing 1-100µM Zol for 10 days. Group of flies were also fed with food containing PBS only (vehicle) for control. (D) Quantification of expression level of pAKT normalised to tubulin (Tub) in presence or absence of Zol (1-100µM) in Drosophila food for 10 days analysed by imageJ (n=3). Data are expressed as mean ±SD and were analysed by one way ANOVA and Sidak post-test for multiple comparisons $^*p<0.05$, $^{**}p<0.01$, $^{***}p<0.001$

eFig. 6

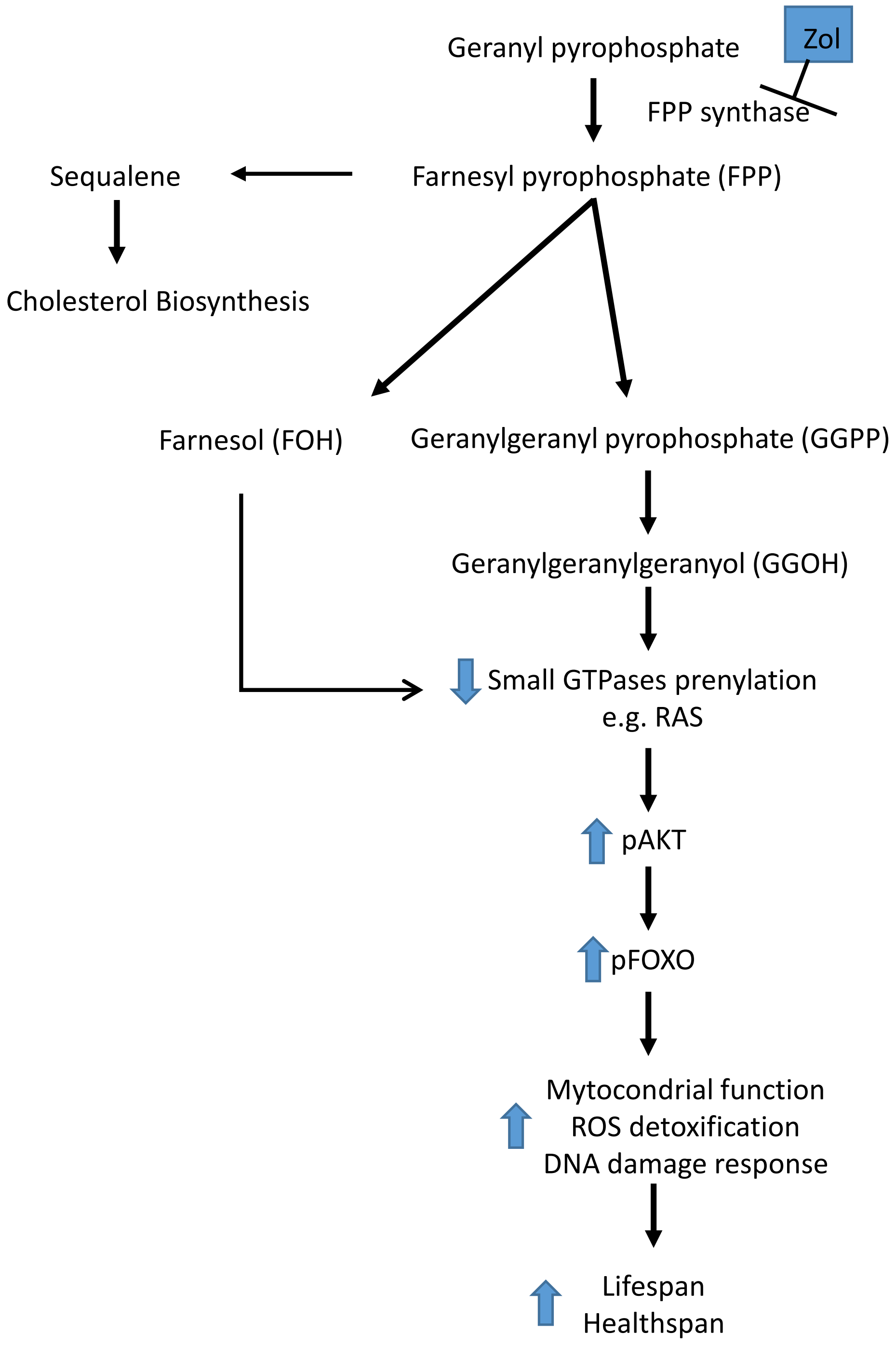

eFig6: Schematic model of signalling events leading to increased lifespan and healthspan by zoledronate
